# Cost-effectiveness analyses of training: a manager’s guide

**DOI:** 10.1186/1478-4491-11-20

**Published:** 2013-05-20

**Authors:** Gabrielle O’Malley, Elliot Marseille, Marcia R Weaver

**Affiliations:** 1 Department of Global Health, International Training and Education Center for Health, University of Washington, 901 Boren, Suite 1100, Seattle, WA, USA; 2 Health Strategies International, 555 59th Street, Oakland, CA, 94609, USA

**Keywords:** Capacity development, Cost-analysis, Cost-effectiveness analysis, Training evaluation, Training effectiveness

## Abstract

The evidence on the cost and cost-effectiveness of global training programs is sparse. This manager’s guide to cost-effectiveness analysis (CEA) is for professionals who want to recognize and support high quality CEA. It focuses on CEA of training in the context of program implementation or rapid program expansion. Cost analysis provides cost per output and CEA provides cost per outcome. The distinction between these two analyses is essential for making good decisions about value. A hypothetical example of a cost analysis compares the cost per trainee of a computer-based anti-retroviral therapy (ART) training to a more intensive ART training. In a CEA of the same example, cost per trainee who met ART clinical performance standards is compared. The cost analysis is misleading when the effectiveness differs across trainings. Two additional hypothetical examples progress from simple to more complex costs and from a narrow to a broader scope: 1) CEA of the cost per ART patient with 95% adherence that compares the performance of doctors to counselors who attend additional training, and 2) CEA of the cost per infant HIV infection averted for a Prevention of Mother to Child Transmission program that compares the current program to one with additional training. To create an evidence base on CEA of training, more well-designed analyses and data on the cost of training are needed. Analysts should understand more about how capacity is built, how quality is improved within a health facility, and the costs associated with them. Considering the life of an investment in training, evaluations are needed on how many trainees apply the skills taught, how long trainees continue to apply them, and how long the content of the training conforms to national or international guidelines. Better data on effectiveness of training is also needed. It is feasible to measure effectiveness by clinical performance standards, or intermediate outcomes and coverage. Intermediate outcomes and coverage can also be combined with published estimates on health outcomes.

## Background

The global response to the HIV epidemic is one of the largest public expenditures to fight a single disease in history [1,2]. This scaled-up response to the HIV epidemic required large numbers of doctors, allied health professionals, and administrative support staff with new, specific HIV clinical and management skills. Recognizing this need, governments, non-governmental organizations, and other international organizations invested heavily in HIV training of health care providers in Africa, Asia, and Latin America. For example, between 2004–2009, as part of the President’s Emergency Plan for AIDS Relief [3] 213,200 health care providers were trained in Prevention of Maternal to Child Transmission (PMTCT) and 304,700 were trained in anti-retroviral therapy (ART).

Global programs for HIV are now moving from emergency response to address sustainability, task-shifting, and integrating HIV care and treatment into primary health care and chronic disease management. This transition will likely involve continuing major investments in training [4,5], and program managers will be looking for good value for these investments. Cost-effectiveness analysis (CEA) is a powerful method to identify value, but many of the well-established methods for conducting these analyses for clinical trials, demonstration projects, and other research have not been applied to scaling-up interventions and the training programs that support it. A notable exception is the WHO Choice Project that estimated the cost-effectiveness of interventions that would contribute to achieving the Millennium Development Goals [6].

Program managers will find evidence is sparse on the cost-effectiveness of global training programs in general [7] and HIV training programs in particular [8]. Schooling and training are investments, known as human capital [9]. CEA of global training to date have focused on how and who to train, as well as training as a component of a larger intervention. Analyses of how to train have addressed the most effective means of training a specific group of health care providers [10-13]. Analyses of who to train have addressed task-shifting or task-sharing in which training was extended to a group of health care providers who assumed new responsibilities for specific services [14-18]. Finally, some analyses have estimated the cost-effectiveness of interventions that included training for the health care providers who implemented them [19-21].

To create an evidence base on the CEA of training, program managers will need to deploy analytic resources wisely. This article has been written as a guide for program managers who may not be economists, but want to recognize and support high quality CEA of training. It focuses on the approaches and challenges associated with conducting CEA of training in the context of program implementation or rapid expansion of programs. For a general consumer’s guide, please see the Panel on Cost-Effectiveness in Health and Medicine’s book [22] or articles [23-25]. The National Library of Medicine offers a free, less technical resource in its online course “Health Economics Information Resources: A Self-Study Course”, which focuses on identifying CEA studies, and appraising their quality [26]. For professionals who plan to conduct CEA, Drummond et al. [27] is a well-respected introduction. In addition, UNAIDS [28] created guidelines with instructions for conducting cost analyses of HIV prevention strategies that can be extended to other HIV programs such as training. These excellent resources, however, predate the scale-up of HIV treatment and care and are oriented to trials, demonstration projects, and other research.

This manager’s guide begins with definitions of cost analysis and CEA. Understanding the distinction between these two analyses is essential for making good decisions about value. Then, three hypothetical examples of CEA of training programs are presented: 1) cost per trainee who met ART clinical performance standards; 2) cost per patient with 95% ART adherence; and 3) cost per HIV infection averted. Each example provides insights on both measures of cost and effectiveness that would contribute to a CEA in the context of program scale-up.

### Cost analysis

A cost analysis estimates the cost per unit of output, where costs are the value of all inputs that a program uses. The inputs typically consist of the time of trainers and trainees, supplies, teaching materials, and possibly equipment, facility space and transportation. Consider a simple hypothetical example in which a program manager is planning to scale-up ART services at a clinic. S/he compares two trainings to upgrade the skills of 25 clinical staff in monitoring patients on ART. One is a computer-based training followed by a three-day practical workshop. The second is an intensive, two-week workshop followed by two on-site trainings. Table 1 presents the cost for the two programs in three categories: 1) training program budget, 2) training venue contract, and 3) ministry of health contribution. For the personnel costs in the training program budget, the remuneration rate, which includes salary and fringe benefits, is multiplied by the number of units (e.g., hours, days, months). The subtotal, which is the sum of the cost for each person, is $7,350 for the computer-based training, and $12,250 for the intensive training.

**Table 1:** **Cost analysis of a computer**-**based training to an intensive training**

	**Computer**-**based training and three**-**day workshop**		**Intensive two**-**week workshop and two on**-**site trainings**		
	**Cost per unit**	**Units**	Cost*	**Units**	Cost*
**Training program budget**					
Curriculum designer	$2,500/month	2	$5,000	2	$5,000
Subject matter experts	$200/hour	9	$1,800	5	$1,000
Trainer	$100/day	3	$300	10	$1,000
Materials: participant manuals	$10/person	25	$250	25	$250
On-site training	$1,000/site	0		5	$5,000
Subtotal training program			$7,350		$12,250
**Training venue contract**					
Rental	$100/day	3	$300	10	$1,000
Meals and tea break	$5/day	75	$375	250	$1,250
Subtotal training venue			$675		$2,250
**Ministry of health contribution**					
Trainees’ time computer-based course	$20/day	125	$2,500		
Trainees’ time workshop	$20/day	75	$1,500	250	$5,000
Trainees’ time on-site training	$20/day		0	25	$500
Subtotal ministry of health			$4,000		$5,500
**Total societal cost**			$12,025		$20,000
**Cost analysis**					
Number of trainees			25		25
Cost per trainee			12025/25= $481		20,000/25= $800

*To calculate each cost, multiply the cost per unit by the number of units. The subtotal is the sum of all the costs in the category.

A cost analysis can differ from a training program budget for at least two reasons: 1) multiple partners contribute to the training, and 2) some contributions are “in-kind.” Training programs are often funded through a combination of contributions by donors, the ministry of health, and the trainees. In Table 1, the cost also includes a training venue contract funded by a donor, and the ministry of health’s contributions. Each partner has a “perspective” and a cost analysis conducted from the perspective of a single partner would focus only on that partner’s budget. A cost analysis conducted from the societal perspective includes the contributions of all the partners as well as trainees and patients. In Table 1, the total societal cost is $12,025 for the computer-based training, and $20,000 for the intensive training.

Some professional guidelines [22,23], recommend that analysts report the cost analysis from societal perspective as a reference case so that costs are comparable across analyses. However, cost analyses could be reported from additional perspectives as appropriate. Program managers should expect analysts to identify the perspective of the analysis and acknowledge that some costs may not be included whenever the cost analysis is not conducted from the societal perspective.

Partners may contribute in-kind as well as with program budgets. In the example in Table 1, the ministry of health contributes the trainees’ time, which should be included in a cost analysis from a societal perspective even if their participation did not require an extra payment to the trainees. The trainees would have engaged in productive work had they not attended the training, which is the *opportunity cost* of trainees’ time. The opportunity cost is the value of the most beneficial alternative feasible use of the resources. Please see the Glossary in Table 2 for definitions of economic terms used in this manager’s guide. This opportunity cost of the trainee’s time is valued at the trainees’ remuneration rate, which is $20 per person per day in this example. In general, an average salary rate for each trainees’ profession, gender and age group is used in the calculation; detailed data on each person’s salary are not necessary.

**Table 2:** Glossary of cost and cost-effectiveness terms

**Annuitization**	Method of allocating the cost of an investment (i.e. a capital cost) over the time period when it is used. When discounting is ignored, annuitization is calculated as the cost of investment divided by the number of years of life of investment.
**Capital Cost**	It is an investment in an asset such as buildings, land, vehicles or equipment that is used over more than one year. The investment is generally a single purchase that may precede the project, so that its cost is not always included in the project budget. The asset has an opportunity cost and its annual cost call be allocated over time.
**Cost analysis**	Estimate of the cost per unit of output, where outputs are the activities or services, such as health care providers trained or patients treated.
**Cost-effectiveness analysis (CEA)**	Analysis of the cost per unit of outcome. The outcome serves as the denominator and must be the same for interventions and programs that are compared. CEA of health and medicine, are generally conducted with health outcomes.
**Cost effectiveness ratio**	The ratio of cost to outcomes. When two programs are not explicitly compared, the ratio represents the cost-effectiveness of the program of interest relative to doing nothing.
**Disability Adjusted Life Years (DALY)**	A measure of the burden of disease of a population in terms of years lost due to ill-health or death. Years of life are weighted from zero to one where zero represents full health and one represents death. Health is characterized by disease categories, and the weights associated with diseases are determined by expert opinion.
**Dominance**	An intervention or program with a lower cost and better outcomes than the alternative.
**Incremental Cost Effectiveness Ratio (ICER)**	The ratio of the difference in cost between two programs and the difference in their effectiveness. The ratio represents the additional cost associated with each additional unit of outcome.
**Opportunity Cost**	The value of the most beneficial alternative feasible use of resources for an activity. For goods and services that are purchased in a competitive market, the opportunity cost is simply the price. Goods and services that are not purchased such as volunteer time should be valued at the cost of purchasing them. Similarly, for goods or services that are subsidized or taxed, they should also be valued at cost to purchase them without the subsidy or tax.
**Perspective**	The point of view from which costs are calculated. Six potential perspectives for a cost analysis of training are: 1) Program perspective might simply be the program budget, 2) Donor perspective would include the budgets of all programs that contribute to an output or outcome, 3) Provider perspective represents the costs of the health care system, including ministry of health, hospitals and clinics, 4) Trainee perspective might be earnings from private practice foregone while attending training, as well as tuition, 5) Patient perspective includes the out-of-pocket expenditures for health care and the time devoted to care for themselves or family members. 6) Societal perspective represents all costs regardless of who bears them including: programs, donors, providers, trainees, and providers.
**Quality Adjusted Life Year (QALY)**	A measure of health of a sample in terms of quality of life years of life lost due to ill-health or death. Years of life are weighted from zero to one where zero represents death or an equivalent state, and one represents full. Health is characterized by health states such as the ability to walk a mile and the weights associated with health states are reported by patients or the community through various research methods.
**Remuneration**	The total amount of resources a consultant or employee receives including salary and fringe benefits.
**Sensitivity analysis**	Calculation of alternative cost-effectiveness results when there is uncertainty about one or more parameters that measure costs or effects. Sensitivity analysis is generally incorporated in CEA that are deterministic, meaning the result is a point estimate. It helps to identify the extent to which uncertainty about a parameter would substantially affect the estimate.

For the cost analysis, the total societal cost is divided by the number of outputs. For training activities, the output produced is typically the number of people trained. In this example, the cost per trainee is $481 for the computer-based training program, and $800 for the intensive training. Program managers should, however, be wary of cost per unit of output. In routine reporting, someone who attended a one-hour lecture might be counted the same way as someone who completed a one-year fellowship, i.e., as a “person trained”. Because of the large difference in resources required and presumably the skills enhancement achieved, an analysis that simply compared the cost per trainee would be misleading. A CEA that measures the cost per outcome can address differences in effectiveness across trainings.

### Cost-effectiveness analysis (CEA)

As applied to health and medicine, a CEA estimates the cost per unit of health outcome such as an HIV infection averted or a year of life gained. The fewer dollars required to achieve an outcome, the more cost-effective the intervention is considered to be. For example, an adult male circumcision program that costs $500 per HIV infection averted is more cost-effective than a voluntary counseling and testing program that costs $5,000 per HIV infection averted.

The scope of a CEA is determined by the denominator; only programs with a common denominator can be compared. For CEA with the broadest scope, the denominator is units of life measured by a combination of mortality and morbidity. Two widely used measures are: QALYs and DALYs. Both measures account for years of life gained from reduced mortality, and improved quality of life during years of reduced morbidity. Both measures also have weights for years of reduced morbidity ranging from zero to one, but the similarities end there. For example, QALYs measure quality of life where death is zero and full health is one, whereas DALYs measure disability where zero is full health and one is death. The measures also differ in objectives, how units of life are characterized, and the methods for calculating the weights associated with each characteristic. Gold et al. provide an excellent comparison between the two measures [29].

As a practical matter, program managers rarely have time or funding to support a CEA that measures the specific contribution of training to health outcomes. Fortunately, an *intermediate measure*, such as the number of health care providers that perform a task competently, or an *intermediate outcome* such as the number of patients who adhere to treatment may be an appropriate denominator for some analyses. It may also be possible to collect original data on an intermediate measure and use published estimates to calculate the health outcomes. These three options are presented in three hypothetical examples below.

### Intermediate measure

Continue with the hypothetical example above in which the computer-based ART training and intensive ART training are compared. The program manager monitors a comprehensive set of costs that extend beyond the training events to include the cost of transfer of learning. As shown in Table 3, the total societal cost of the computer-based training is $12,025 and of the intensive training is $20,000 as in Table 1. The cost analysis in Table 3 also includes the time senior staff spent with graduates after the training to support them to apply their new skills, such as ad hoc sessions in the context of actual patient visits. This cost of the supervisors’ time in providing this additional support is $8,000 among graduates of the computer-based training versus $2,000 among graduates of the intensive training. The total cost of the training and transfer of learning is $20,025 for the computer-based training and $22,000 for the intensive training.

**Table 3:** **CEA with an intermediate outcome**: **cost per trainee who meets ART clinical performance standard**

	**Computer**-**based training and three**-**day workshop**	**Intensive two**-**week workshop and two on**-**site trainings**
Cost of transfer of learning*		
Cost of training	$12,025	$20,000
Cost of follow-up support by supervisors	$8,000	$2,000
Total cost of transfer of learning	$20,025	$22,000
**Cost analysis**		
Trainees	25	25
Cost per trainee	$20,025/25 = $801	$22,000/25 = $880
**Cost**-**effectiveness analysis**		
Trainees who meets ART clinical performance standard**	15	22
Cost per trainee who meet ART clinical performance standard	$20,025/12 = $1335	$22,000/23 = $1000

*Transfer of learning includes both the training events and follow-up support.

**The ART clinical performance standard is to perform 80% of the task correctly. According to this measure, the computer-based training was less effective.

The program manager selects a set of tasks to measure clinical performance to compare the effects of the trainings. A graduate who performs 80% of the tasks correctly meets the standard. In spite of post-training support, only 15 graduates of the computer-based training meet the standard, whereas 22 graduates of the intensive training do. Each training is compared to no training, i.e., doing nothing, so the cost-effectiveness ratio is simply the total societal cost divided by the number of trainees who meet the standard. As shown in Table 3, the cost per successfully trained graduate of the computer-based training is $1,335 versus $1,000 for the intensive training.

The key to this result is collecting cost data on all the activities associated with transfer of learning and measuring effectiveness in terms of clinical performance. If success was measured simply by the number of trainees as in Table 1, the lower cost per graduate might displace competence as the standard for selecting a training.

### Intermediate health outcomes

Intermediate health outcomes are used in a CEA with a somewhat broader scope. Consider an agency that is committed to providing ART to HIV-infected adults and children. Since the health benefits of ART are well-documented [30-33], a program manager might want to focus on extending coverage and improving adherence. The program manager’s question in this instance is “How can I allocate my budget to provide high quality care to the most patients?” This broader scope supports comparison of training programs to other activities that may affect coverage and adherence, such as pharmacy logistics, continuous quality improvement, or leadership initiatives.

In this hypothetical example (Table 4), a clinic is providing ART in an impoverished urban setting where many patients have substance abuse problems, or other challenges to adherence. While good adherence is not a health outcome, studies showed that the correlation between good adherence and viral suppression is high [34-38]. The program manager adopts an intermediate outcome; the number of patients maintaining 95% adherence. Some experts argue that setting a high standard for adherence is discouraging to patients, and alternative levels such as 80% or simply mean adherence among patients could also be used in an analysis [35].

**Table 4:** **CEA with an intermediate health outcome**: **cost per patient with 95**% **ART adherence**

	**Pre**-**training**	**Post**-**training**
**Program cost**		
Remuneration	$50,000	$30,000
Medication	$100,000	$110,000
Total program cost	$150,000	$140,000
Cost of training (annuitized)*	$0	$5,000
Total cost	$150,000	145,000
**Cost**-**effectiveness analysis**		
Number of patients	400	440
Percentage of patients with 95% ART adherence	90%	85%
Number of patients with 95% ART adherence	360	374
Cost-effectiveness result		Dominates**

*The annual cost of a $20,000 training with a four-year life of investment is $5,000.

**No cost-effectiveness ratio is necessary, because a program with lower cost and higher effectiveness dominates the alternative.

Prior to training, relatively highly-paid doctors spend much of their time screening patients at risk for poor adherence, counseling them, and referring them for additional support. As shown in the Pre-training column of Table 4, ART services cost $150,000 and served 400 patients each year. Adherence is 95% among 360 patients (90%) at a cost of $417 per patient with 95% adherence.

The program manager sees an opportunity to increase coverage through task-shifting or task-sharing and funded training for counselors to assume responsibility for the screening, counseling, and referral activities. In this example, as the measure of effectiveness shifts from trainees to patients, the benefits of an investment in training last several years when trained staff continue to apply their new skills. The annual measures of patient outcomes are compared to the annual cost of the investment in training. Distributing the cost of an investment over time is called “annuitization.” Just as the use of a $40,000 automobile can extend for 10 years at a cost of $4,000 per year, the total cost of training, say $20,000 in this example, that extends for four years is $5,000 per year (for simplicity, discounting is ignored in these examples.) The annual cost depends on how many trainees apply the skills learned, how long they apply them, and how long the new skills remain the standard of care. For example, Hiner et al. reported that 65% of trainees provided voluntary counseling and testing in the Caribbean three years after training on these services [39]. Namagembe et al. reported that improved clinical practice persisted for at least one year after a malaria case management training program [40]. Research in the United States showed that 50% of practice guidelines were outdated in 5.8 years [41].

As shown in the Post-training column, the annual training cost is $5,000. There are other changes in cost after the training. Personnel costs decrease from $50,000 to $30,000 as doctors with high salaries are replaced by counselors. The trained counselors move patients through the ART screening, counseling, and referral process more quickly, which increases the number of patients on ART from 400 to 440. Some of the savings in personnel costs are offset by the cost of medications for 40 additional patients. Total program cost decreases from $150,000 to $140,000, which would not be the case if the total cost of training were allocated to the first year.

The quality of the new screening, counseling, and referral activities is, however, lower than before the training; the counselors are less effective than doctors. Adherence is 95% for 374 of 440 patients (85%). Nevertheless, the expanded coverage means that more patients achieve 95% adherence post training than before. The training dominates the alternative, because the total cost is lower and number of patients with 95% adherence is higher post training than before. The key to this result is tracking service volume to show the training is more cost-effective than if the CEA focused only on adherence.

### Health outcomes

The hypothetical example in Table 5 combines data on coverage with published estimates of the health outcomes associated with PMTCT. Before an intervention is scaled-up in HIV care, there is generally extensive research on its effectiveness. It is neither feasible nor necessary for each training to replicate the earlier research. It is sometimes possible to apply the results of earlier research to estimate the effects of training on health outcomes. The CEA Registry is a good resource for identifying previous research on cost-effectiveness [42].

**Table 5:** **CEA with a health outcome**: **cost per HIV infection averted**

	**Pre**-**training**	**Post**-**training**
**Program cost**		
***Remuneration***		
Clinical staff, including counselors	$65,000	$72,000
Supervisory staff	$15,000	$12,000
Total remuneration	$80,000	$84,000
**Supplies, including HIV test kits and ARV drugs**	$15,000	$18,000
***Capital (annuitized)***		
Vehicle	$3,000	$3,000
Equipment	$500	$500
Building	$1,500	$1,500
Training	$0	$5,000
Total Capital	$5,000	$10,000
Total program cost	$100,000	$112,000
**Estimate of effectiveness**		
Number of mother infant pairs that PMTCT	1000	1200
Estimated vertical HIV transmission before and at birth*		
Base case	25%	25%
Lower bound	19%	19%
Upper bound	30%	30%
Effectiveness of regimen for mothers and infant	63%	63%
***Estimated HIV infections averted***		
Base case	(1000*.25*.63) =158	(1000*.25*.63) =189
Lower bound	120	144
Upper bound	189	227
**Cost**-**effectiveness analysis**		
Incremental cost	($112,000-$100,000) = $12,000	
***Incremental HIV infection averted***		
Base case		(189–158) = 31
Lower bound		(144–120) = 24
Upper bound		(227–189) = 38
***Incremental cost***-***effectiveness ratio*** (***ICER***)		
Base case		($12,000/31)=$283
Lower bound		$215
Upper bound		$340

*The effect of the training on MTCT is uncertain because there is a range of estimates for vertical HIV transmission before and during birth. The midpoint of the range is used to estimate effectiveness for the base case, and a sensitivity analysis is conducted with the lower and upper bounds. The base case and range are also reported for the number of HIV infections averted and ICER.

Even the best research, however, may not provide a single estimate of costs or effects. Results from a single study are reported as a mean and confidence interval, and results may not be identical across multiple studies. When there is uncertainty about parameter estimates, analysts conduct a sensitivity analysis with a base case and alternative estimates that show a range of cost-effectiveness ratios. Uncertainty may exist for a variety of reasons and this example demonstrates a sensitivity analysis for estimates of health outcomes.

Considering PMTCT, HIV is transmitted during antenatal, delivery, and breastfeeding periods, but it is not possible to identify precisely the transmission associated with each period with available HIV testing methods. Research in the 1990’s showed that, in the absence of treatment, HIV transmission in the antenatal or intrapartum period ranged from 19% to 30% and transmission during breastfeeding ranged from 10% to 16% [43]. More precise estimates may never exist, because the earlier studies proved that treatments were effective and it would now be unethical to withhold treatment. Consequently, a CEA would present a sensitivity analysis of the effect of a PMTCT training on HIV infections averted.

Begin with the cost analysis of the PMTCT program in a district hospital that cost $100,000 per year. The program manager wants to know if money spent on counselors’ training is well spent. If so, s/he will seek funding to extend this training to other PMTCT sites. Before the training, costs are apportioned across personnel, medications and capital categories as shown in Table 5. Personnel costs are calculated in two categories: 1) clinic staff, including counselors, and 2) supervisory staff. For clinic staff, the number of full-time equivalent (FTE) staff providing counseling, HIV testing, and ART screening and treatment is multiplied by their remuneration rate per FTE for a total of $65,000. For supervisory staff, the portion of their time spent supporting PMTCT is multiplied by their remuneration rate for a total of $15,000. For supplies, $15,000 is spent on HIV test kits, ARV drugs, and other supplies. Capital costs are calculated for three categories, each annuitized to an annual cost: 1) transportation is $3,000, 2) equipment such as refrigerators, computers and furniture is $500, and 3) building is $1,500. The ministry of health provides the hospital building used for PMTCT free-of-charge. Nevertheless, absent PMTCT, the space could be used for other worthwhile activities. Its opportunity cost is approximated by the annual rent for equivalent space if procured on the market.

For the pre-training effectiveness, the program serves 1,000 women and infant pairs per year who complete the ARV regimen. The number of infant HIV infections averted each year is estimated by multiplying the number of women and infant pairs by the published antenatal and intrapartum transmission rates [43] and published estimate of 63% efficacy for the PMTCT regimen provided at the clinic [44]. For the antenatal and intrapartum transmission rates, the base case uses the midpoint of the range of estimates (25%), and the sensitivity analysis uses the lower (19%) and upper (30%) bounds. As shown in Table 5, the base case estimate of the number of HIV infections averted is 158 with a range from 120 to 189.

Post-training, the cost changes in important ways. Because the counseling protocol requires longer counseling sessions and smaller group sizes, the personnel cost of clinical staff, including counselors, increases from $65,000 to $72,000 annually. The training includes routine record keeping, however, and allows senior nurses to take on supervisory functions formerly left to more highly paid supervisors. The result is a reduction in personnel costs of supervisors from $15,000 to $12,000 per year. The cost of supplies increases from $15,000 to $17,000, because more women and infants complete the PMTCT sequence and consequently use test kits and ARV drugs. The training program cost $20,000 and it is estimated to have four years of benefit before skills degrade or staff members leave, with an annual cost of $5,000 per year.

For the post-training effectiveness, the number of women and infant pairs who complete the PMTCT regimen increases to 1,200. The base case estimate of the number of HIV infections averted is 189 with a range from 144 to 227. Both cost and HIV infections averted increased. The incremental cost-effectiveness ratio is the increase in total cost ($112,000 – $100,000) divided by the increase in infections averted (189 – 158), which is $283 per HIV infection averted with a range from $215 to $340.

## Conclusions

The cost data included in a cost analysis depend on its perspective. Some professional guidelines recommend that published cost analysis report the societal perspective, which include the cost of all partners, as well as trainees and patients. Cost analyses can, however, be misleading when the effectiveness of the trainings differ.

A CEA can guide program managers to make the best investments in training. The three hypothetical examples progressed from simple to complex costs and a narrow to broader scope to highlight key concepts about measuring the cost and effects of training in the context of program implementation or rapid expansion.

To create an evidence base on CEA of training, the examples in this manager’s guide demonstrate the need for more well-designed analyses and data on the cost of training. Analysts should understand more about how capacity is built, how quality is improved within a health facility, and the costs associated with them. The analyses can then collect data on how the cost of capacity-building and quality improvement change in response to training. Considering the life of an investment in training, analysts should focus on how many trainees apply skills taught during the training, how long trainees continue to apply them, and how long the content of the training conforms to national or international guidelines.

The examples also demonstrate the need for better data on effectiveness of training programs. It is feasible to conduct CEA of training in which effectiveness is measured by clinical performance standards, or intermediate outcomes and coverage. Intermediate outcomes and coverage can also be combined with published estimates on health outcomes to estimate the effect of training on health outcomes. Program managers are encouraged to maintain a focus on these effectiveness measures rather than outputs and to invest in trainings with the lowest cost per improvement in them.

## Authors’ contributions

GO led manuscript conceptualization and outline, conducted literature review, and edited multiple manuscript iterations including contributing original text. EM provided substantial edits and contributed original text. MW wrote early drafts and substantial edits, and reviewed final manuscript. All authors read and approved the final manuscript.

## Sources of support

This study and the activities detailed were developed and conducted by the University of Washington and I-TECH with funding from Cooperative Agreement U91HA06801-06-00 from the US Department of Health and Human Services, Health Resources and Services Administration (HRSA).
